# COVID-19 outbreak impact in Spain: A role for tobacco smoking?

**DOI:** 10.18332/tid/120005

**Published:** 2020-04-06

**Authors:** Javier C. Vázquez, Diego Redolar-Ripoll

**Affiliations:** 1 NutriNeuro, Bordeaux Neurocampus, Bordeaux, France; 2 Cognitive NeuroLab, Faculty of Health Sciences, Universitat Oberta de Catalunya, Barcelona, Spain

**Keywords:** smoking, tobacco, COVID-19, coronavirus, sex differences

**Dear Editor,**

Coronavirus disease 2019 (COVID-19) is an infectious disease provoked by severe acute respiratory syndrome coronavirus 2 (SARS-CoV-2)^1^. The virus typically spreads from person to person via respiratory droplets produced during coughing, and common symptoms include fever, cough, and shortness of breath^2^. The COVID-19 outbreak was initially identified in Wuhan, capital of Hubei Province, China, in December 2019, and has since spread rapidly globally. It was declared a pandemic on 11 March 2020 by the World Health Organization (WHO)^3^. As of 26 March 2020, a total number of 480446 cumulative cases of COVID-19 have been reported in 175 countries and regions, including 22030 confirmed deaths, the majority of which have been reported in Italy (7503), Spain (4089) and China (3169)^4^. In Spain, as of 25 March 2020, most of those who have died were elderly, about 96% of deaths were in those over 60 years old, and 45% had pre-existing health conditions including cardiovascular disease (31%)^5^.

With 120859 deaths in 2018, cardiovascular disease (CVD) is the leading cause of death in Spain (28.3%)^6^. Sex-disaggregated data for CVD in Spain show differences in mortality between men (46.3%) and women (53.7%)^6^. Surprisingly, although men (51%) and women (49%) are getting infected by COVID-19 at similar rates, men have been dying from COVID-19 at a significantly higher rate (4.4%) than women (2.5%), and the sex differences regarding vulnerability in those with COVID-19 and preexisting CVD seem to be again reflected in men (35%) and women (26%)^5^.

Emerging evidence suggests that these discrepancies could potentially be due to gendered differences such as patterns and prevalence of smoking. In this regard, approximately 10% of cardiovascular disease is globally attributed to smoking^7^, with smoking prevalence in 2017 among men in Spain being approximately 25.6% but only 18.8% in women^8^.

Could smoking influence the gender-based impact of the outbreak? And the impact itself?

These hypotheses could be coupled with new available evidence from the WHO on COVID-19, warning that a weaker cardiovascular system among COVID-19 patients with a history of tobacco use could make such patients susceptible to severe symptoms, thereby increasing the chance of death^9^. According to the recent (18 March 2020) systematic review of Vardavas and Nikitara^10^, smoking is most likely associated with the negative progression and adverse outcomes of COVID-19.

Accordingly, we recommend that public health messages and behavioural interventions coming from the Spanish government that focus on how to spread and flatten the COVID-19 infection curve should also consider available evidence-based high-quality smoking cessation advice.
